# Prevalence of short stature and malnutrition among Egyptian primary school children and their coexistence with Anemia

**DOI:** 10.1186/s13052-020-00855-y

**Published:** 2020-06-29

**Authors:** Ali M. El-Shafie, Zeinab A. Kasemy, Zein A. Omar, Safa H. Alkalash, Amal A. Salama, Kerollos S. Mahrous, Shaimaa M. Hewedy, Nessreen M. Kotb, Heba S. Abd El-Hady, Eman S. Eladawy, Mohamed A. Zeid, Manar E. Abd El Hamid, Emad H. Hemeda, Mohamed A. El-shafie, Esraa A. El-Meligy, Wael A. Bahbah

**Affiliations:** 1 Department of Pediatrics, Faculty of Medicine, Menoufia University, Shebin El-Koum, Egypt; 2 Department of Public Health and Community Medicine, Faculty of Medicine, Menoufia University, Shebin El-Koum, Egypt; 3 Family Medicine Department, Faculty of Medicine, Menoufia University, Shebin El-Koum, Egypt; 4 Shebin El-Koum, Menoufia, Egypt; 5 Ministry of Health Hospitals, Cairo, Egypt

**Keywords:** Anemia, Egyptian children, Malnutrition, Obesity, Prevalence, Short stature

## Abstract

**Background:**

Under nutrition and overweight typically occur during nutritional transition periods in developing countries including Egypt. Short stature and anemia are public health concern due to its strong link with malnutrition which is a preventable risk factor.

**Objectives:**

to estimate the prevalence of overweight, obesity, underweight and short stature and its concurrence with anemia, also to determine the etiological profile of short stature among primary school children in Egypt.

**Methods:**

A cross-sectional study was carried out on 33,150 Egyptian children aged 6–11 years old from January 2018 to January 2020, allocated in 59 primary schools from diverse geographical districts in Egypt. Complete anthropometric measurements were conducted and applied according to WHO growth charts. Hemoglobin level was measured. Systematic approach to detect the etiology of short stature was applied randomly to a sample of 380 stunted children.

**Results:**

The prevalence of underweight was 8.2%, while obesity and overweight represented 21.8% (9.6 and 12.2% respectively). Overall short stature constituted 17%. The main etiologies of short stature were familial (40.8%) and constitutional (24.2%). Anemia was diagnosed in 26% of children; while concurrent anemia and stunting was reported in 9.9%. Regarding anemia and anemia with stunting were more common among girls (30.0% (OR = 1.50, CI95%: 1.43–1.58) and 11.4% (OR = 1.39, CI95%:1.29–1.49) respectively), who were living in rural areas (33.4% (OR = 1.96, CI 95%:1.87–2.06) &12.7% (OR = 1.72, CI 95%:1.60–1.85)) and those who had low socioeconomic status)34.6% (OR = 2.54, CI 95%:2.29–2.82) & 17.2% (OR = 3.32, CI 95%:2.85–3.88() respectively. Anemia with stunting was significantly higher among children aged ≥9 years old representing 12% (OR = 1.40, CI 95%:1.30–1.51).

**Conclusion:**

Prevalence of short stature, obesity and anemia was high among primary school children in Egypt with a strong concurrence between anemia and stunting. Intensive parental health education and in-depth nutritional assessment are required.

## Background

The double burden of malnutrition is defined by coexistence of both under nutrition and overweight with increasing risk of non-communicable diet related diseases [1]. It is a global challenge affecting all over world countries with higher burden on underdeveloped and developing countries [2]. The causes may be related to a sequence of epidemiological changes known as nutritional, epidemiological and demographic transition and it confers a negative impact on economy of individuals and populations [3, 4]. In Egypt two thirds of under 5 years child mortality owed to malnutrition and stands as one of the 36 countries where 90% of the global burden of malnutrition falls [5]. Pediatric overweight and obesity are considered the most prevalent nutritional disorder among both children and adolescents with 21–24% of them are overweight [6]. They increase the risk of heart diseases, insulin resistance and diabetes (type 2), hyperlipidemia, hypertension, kidney and liver diseases and reproductive dysfunction [6]. WHO has reported rapid increase in their prevalence among all pediatric age groups [7]. Short stature is defined as a height that is 2 standard deviations (SD) or more below the mean height for individuals of the same sex and chronologic age in a given population [8]. Short stature, also called stunting frequently misinterpreted as a proxy indicator for malnutrition; malnutrition may lead to stunting, but stunting itself does not indicate malnutrition [9, 10]. Most children with short stature have normal variants such as familial short stature, constitutional delay of growth and puberty, or idiopathic short stature while the most common pathological etiologies are growth hormone deficiency (GHD), hypothyroidism, celiac disease, and Turner syndrome, other causes include renal, hepatic, and gastrointestinal diseases, and other genetic syndrome [11]. Stunting often goes unrecognized in communities where short stature is the norm; On the other hand it may be the first presentation of underlying disease or health problem and it is considered a cyclical process, creating an intergenerational cycle of poverty that is difficult to break [12, 13]. Underweight in a child with short stature suggests a systemic illness or malnutrition, whereas overweight suggests an endocrine disorder; if the initial evaluation suggests a genetic, endocrine, or gastrointestinal disorder [14]. Anemia is a global problem affecting around 305 million school children aged 5–15 years and it is 3–4 times more prevalent in non-industrialized regions than industrialized ones [15]. In spite of high correlation between anemia and nutrient deficiency which represents more than half of causes, but different varieties of hemoglobinopathies and chronic hemolytic anemia are worthy of research especially in geographic areas of high frequency [15, 16]. As each of anemia and stunting represent a significant burden and challenge to the health system of children, their concurrence would be even more disastrous [17]. The aim of this work was to estimate the prevalence of overweight, obesity, underweight and short stature and its concurrence with anemia, also to determine the etiological profile of short stature among primary school children in Egypt.

## Methods

**Fig. 1:**
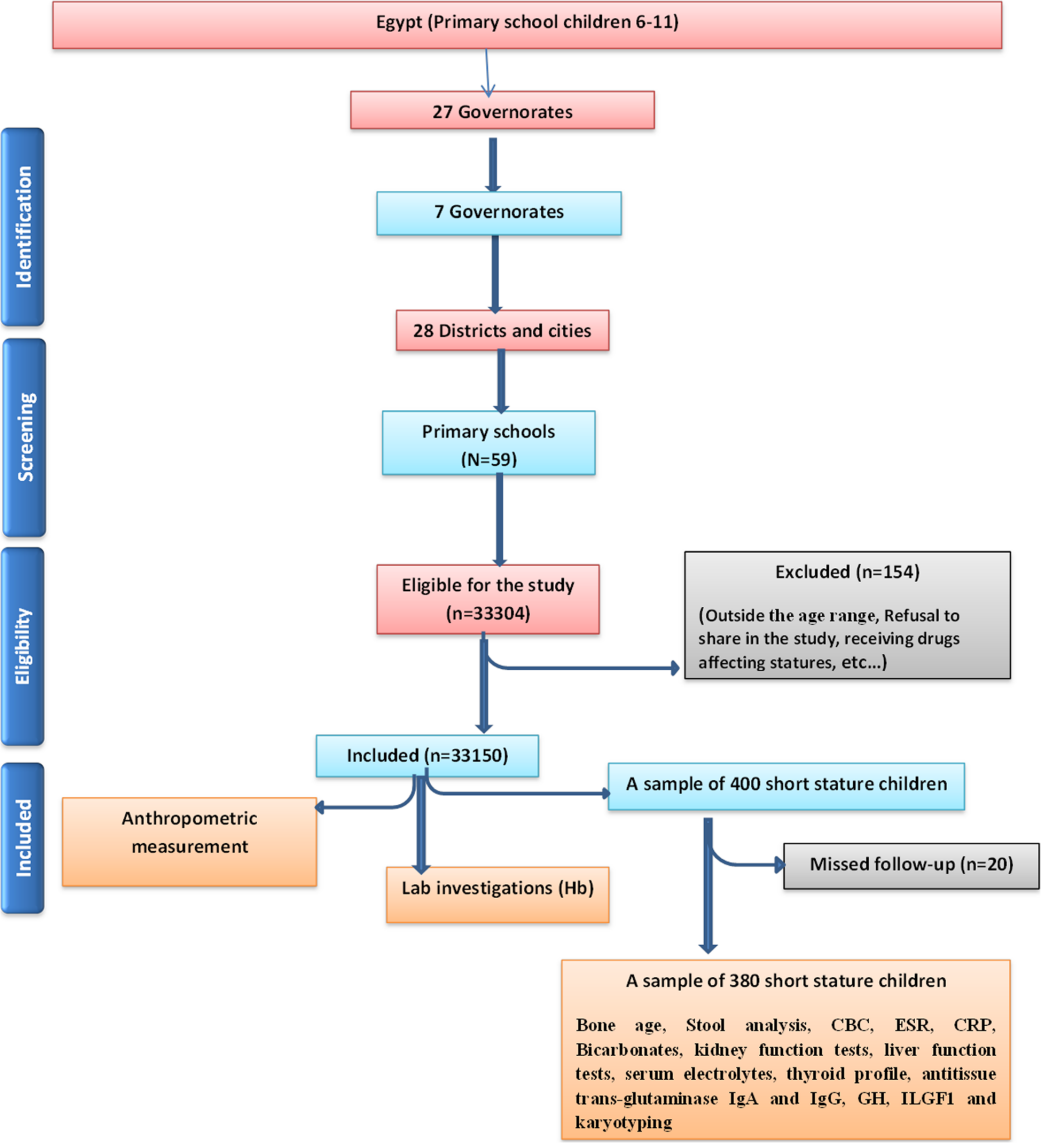
Flow chart of sampling

A multistage random sampling technique was used to randomly select a cross-sectional study sample from a stratified listing of Egyptian primary school children aged 6–11 years, available at the time of study design. The study sample was determined based on the Egypt demographic health survey 2015 [18]. All details of the selected schools in cities and villages in every single governorate were provided. The process was totally computerized. A total national sample of 33,354 children was eligible for the study. The exclusion criteria were applied to 154 children leaving 33,150 children as a final total sample to be included in the study. The study was carried out in a time frame from the beginning of January 2018 till the end of January 2020 at 59 primary schools. (Figure 1) All types of schools (public, private and language schools) were included. All the socioeconomic strata were represented with weighted rural-urban representation. Two full-days’ workshop training was provided to field work team consisted, primary care physicians, pediatricians and nurses. Workshop training was conducted in each of the chosen 7 governorates of Egypt with providing more sessions to the teams of large regions. A pilot study of 50 children was designed to test all items of the project and also to test and standardize the capabilities of the involved team before proceeding to the main data collection. The field work included the school health supervisor, field team supervisors, doctors and nurses. The trained team made visits to all selected schools to complete a socio-demographic questionnaire, provide clinical examination and take body measurements. Children were sent to a chosen standardized laboratory center in every studied region to provide blood and stool sample to be examined.

### Inclusion and exclusion criteria

Recruitment for the study was based on following inclusion criteria: Egyptian children of both sexes, aged from 6 to 11 years. The exclusion criteria were: children less than 6 years or more than 11 years old, children whose mothers/guardians or caregivers refused medical examination and investigation, receiving drugs known to cause short stature including chronic use of steroids, attention-deficit/hyperactivity disorder medications and anticonvulsants during the time of examination.

The mother/guardian or caregiver of each participating child was interviewed for potential determinants of child nutritional status including socio-demographic characteristics. Socioeconomic standard (SES) was assessed by updated scaling score of SES developed by Fahmy et al., 2015 [19]. Physical assessment and hemoglobin (Hb) level assessment were carried out to all children.

### Measurements

Each child was instructed to stand on the center of a digital balanced scale (Beurer model GS 11, Germany) for measuring the weight and height assessed by Harpenden fixed stadiometer. Weight and height of each child was measured after calibrating to the nearest 0.1 kg and 0.1 cm, respectively. Horizontal Frankfurt plane with occiput, shoulder, buttocks, and heel touching the wall and the arms was down and relaxed. Each child was measured while wearing light clothes after removing shoes, belt, cap, hair style or any other material that could interfere with their actual height and weight. Body mass index (BMI) was calculated according to equation “(weight (in kg)/height squared (m2)”. Each of the three measurements was applied to WHO 2007 standard deviation growth charts [20]. Anthropometric data on the height for age of children is used to quantify stunting. A child is stunted if their height for age Z score (HAZ) is < 2 SD below the WHO median where the HAZ of child i is measured as HAZij = hij − h – j/ σj where hij is the height of child i in group j; where the group is based on the child’s sex and age. h − j is the median height of group j in the new WHO reference population and σj is the standard deviation value of group j in the new WHO reference population [20]. Two dates were so important for the determination of the exact age at the time of measurement, the exact birth date (day/month/year) from an official document and the exact date of body measurements.

### Laboratory investigations

Students were subjected for anemia screening using the HemoCue (HemoCue® Hb 201+) photometer. Hemoglobin concentration was read to the nearest 0.1 g/dL. Children with blood hemoglobin concentration level < 11.5 g/dL were considered anemic according to age specific cut off levels established by WHO [21].

### Assessment of stunted children

Based on a past review of literature reported that the percentage of familial cause of stunting was 38% [22]. Sample size has been calculated using the following equation: *n* = (z^2^ × p × q)/ D2 at CI 95% and it was 356. To avoid drop out, 400 children with short stature were selected randomly for follow-up for a year. Twenty stunted children were missed during follow-up leaving 380 children. A specific approach inspired from consensus of European Society for Pediatric Endocrinology 2008 was followed to assess the etiological profile of stunting [8]. A high specialty center was chosen to ensure presence of all required laboratory investigations and to avoid annoying the children by stressful transportation to many places. The initial evaluation included an accurate height measurement, detailed medical and nutritional history, birth weight, physical examination in addition to family history of short stature and pubertal age of both parents. Target height was calculated by the method of mid-parental height, the average of the mother’s and father’s height ± 6.5 cm (addition in boys or subtractions in girls) [8]. Then calculation of the growth velocity was performed after 1 year follow up of their height, which considered normal when it is at least 4 cm per year [23]. All stunted children were investigated for bone age by radiography, and then assessed by radiologist according to published standards of Greulich and Pyle’s atlas of skeletal development [24]. Stunted children who had decreased growth velocity, symptoms of pathological disorders or severely stunted were investigated for: Stool analysis, complete blood count (CBC), erythrocyte sedimentation rate (ESR), C-reactive protein (CRP), kidney function tests, liver function tests, serum electrolytes (calcium, phosphorus and alkaline phosphatase), bicarbonate, thyroid profile (Thyroid stimulating hormone (TSH), free thyroxin (T4)) and anti-tissue trans-glutaminase IgA and IgG (cases which tested positive were confirmed by endoscopic biopsy for celiac disease). After exclusion of other causes of short stature and systemic diseases with high clinical suspicion of GHD (height < − 3 S. D from mean or height ≤ 2 S. D of the corrected mid-parental height and delayed maturation of bone), Growth hormone (GH) levels (after appropriate provocation test) and insulin like growth factor-1 (IGF-1) were investigated. GHD was diagnosed when GH peak level is under 10 ng/l and low IGF-1 [23]. Karyotyping was done for severe stunted female (<− 3 SD.) without other causes even if no specific stigmata for Turner syndrome [8]. Idiopathic cause was diagnosed when child with short stature, a subnormal growth velocity, delayed bone age, no other medical cause, and normal provocative test of growth hormone [25].

Only data relevant to each child were entered in record then questionnaires containing socio-demographic data together with added laboratory investigations and findings of clinical examination were reviewed for completeness by the investigators. Dealing with any missing, unclear or incomplete data were done accordingly with the regional and field supervisor.

### Patient and public involvement

This work aimed to screen the prevalence of short stature, malnutrion and anemia among Egyptian primary school children. To improve the relevance of research, research oriented to mothers and the public. The interviewed mothers recommended generalizing the screening over a large population and conducting it in the most crowded districts. Hence we asked them to tell every mother in their residence areas to communicate with their regional family health facilities and asking for simple growth monitoring of their children to early detect if there are any deviation in their children growth to be early corrected. A special day per governorate had been organized to thank all participants, disseminate the results and provide an in-depth group health education session. For other mothers, the main aim of the health education session was to correct wrong information and to build knowledge base for new mothers so they know well basics of proper nutrition.

### Statistical analysis

Analysis of data was done using the Statistical Package for Social Sciences™ (Version 22.0; IBM Corp., Armonk, NY, USA). Frequency was used to describe the characteristics of the study population and to estimate the prevalence of underweight, overweight, obesity, anemia and short stature. Chi-square test was used for categorical variables. OR = ad/bc was applied to assess degree of risk where OR = 1➔nil, OR > 1➔risky exposure, OR < 1➔ Protective exposure. *P*-value < 0.05 was set to be significant.

## Results

A total national sample of 33,150 children aged 6–11 years old were enrolled in the study with nearly equal male and female percentage. Other demographic characteristics of the studied participants were included in Table 1. BMI was calculated and the results revealed that Overweight and obesity represented 21.8% (9.6% obese and 12.2% overweight) while 8.2% of the children were underweight. Height of the participants was measured and the results showed that short stature was reported among 17% of the studied children while tall stature was reported in 3.9%. With studying prevalence of short stature among obese children it was found that only 0.9% of them showed stunted growth. The current study revealed that prevalence of anemia among primary school children was 26% while anemia with stunting was reported among 9.9% of the total studied sample (which accounted of 58.1% of stunted children).

**Table 1:** Demographic characteristics of the studied participants

	Participants (No. = 33,150)	
	no	%
**Age(Y)**		
< 9	21,989	66.3
≥ 9	11,161	33.7
**Sex**		
Male	17,143	51.7
Female	16,007	48.3
**Residence**		
Urban	18,858	56.9
Rural	14,292	43.1
**SES**		
Low	4315	13.1
Moderate	24,970	75.3
High	3865	11.6

The etiology of short stature was distributed as; non pathological causes [familial (40.8%), constitutional (24.2%), idiopathic (6.6%)], Malnutrition (6.8%), endocrinal causes [Hypothyroidism (7.6%), growth hormone deficiency (9.7%)], Systemic non endocrinal causes [(celiac disease (3.4%), chronic kidney disease (0.5%)] and Turner Syndrome (0.3%). (Table 2) On studying distribution of height and BMI regarding demographic data among the studied participants; it was shown that there were highly statistically significant differences between both of them and children age, sex, residency and their socioeconomic status as it was found that 25.2% of children aged ≥9 years were stunted and 25.4% of children ≥9 years were overweight/obese, male children showed more stunting (19.2%) and females were more overweight (25.8%), also children from rural areas recorded more percentages of stunted growth (18.3%) while (27.1%) of urban children showed overweight/obesity. Low SES was associated with stunting (29.7%) while high SES was associated with overweight and obesity (25.5%). (Table 3) Regarding distribution of anemia and anemia with stunting in relation to demographic data among the studied participants, there was statistically significant differences as regards children age, sex, residency and their socioeconomic status. Regarding anemia and anemia with stunting were more common among girls (30.0% (OR = 1.50, CI95%: 1.43–1.58) and 11.4% (OR = 1.39, CI95%:1.29–1.49) respectively), who were living in rural areas (33.4% (OR = 1.96, CI 95%:1.87–2.06) &12.7% (OR = 1.72, CI 95%:1.60–1.85)) and those who had low socioeconomic status)34.6% (OR = 2.54, CI 95%:2.29–2.82) & 17.2% (OR = 3.32, CI 95%:2.85–3.88() respectively. Children aged < 9 years old showed higher prevalence of anemia 27.3% (OR = 1.23, CI 95%:1.17–1.30) while anemia with stunting was significantly higher among children aged ≥9 years old representing 12% (OR = 1.40, CI 95%:1.30–1.51) (Table 4).

**Table 2:** Etiology of short suture among studied children (*N* = 380)

Etiology of short suture	*N* = 380	%
Non pathological variants:		
▪Familial	155	40.8
▪Constitutional	92	24.2
▪idiopathic	25	6.6
Endocrinal causes:		
▪GHD^b^	37	9.7
▪Hypothyroidism	29	7.6
▪Malnutrition	26	6.8
Systemic non endocrinal causes:		
▪Celiac disease	13	3.4
▪CKD^a^	2	0.5
▪Turner syndrome	1	0.3

^a^*CKD* chronic kidney disease, ^b^*GHD* growth hormone deficiency

**Table 3:** Distribution of height and BMI regarding demographic data among the studied participants

	Height						***P*** value	BMI						***P*** value
	Short suture (No. = 5651)		Normal (No. = 26,221)		Tall suture (No. = 1278)			Underweight (No. = 2707)		Normal (No. = 23,213)		Overweight/ obese (No. = 7230)		
**Age (Y)** < 9 (*n* = 21,989)	**no**	**%**	**no**	**%**	**no**	**%**	< 0.001*	**no**	**%**	**No**	**%**	**no**	**%**	< 0.001*
	3465	15.8	18,091	82.3	433	1.9		1639	7.5	15,953	72.5	4397	20.0	
≥9 (*n* = 11,161)	2186	25.2	8130	74.5	845	7.6		1068	9.6	7260	65.0	2833	25.4	
**Sex**														
M (*n* = 17,143)	3343	19.2	13,657	79.7	143	0.8	< 0.001*	2089	12.1	11,959	69.8	3095	18.1	< 0.001*
F **(*n*** **= 16,007)**	2308	14.4	12,564	78.2	1135	7.0		618	3.9	11,254	70.3	4135	25.8	
**Residence**														
U (*n* = 18,858)	3029	16.1	15,064	79.9	765	4.0	< 0.001*	1006	5.3	12,738	67.5	5114	27.1	< 0.001*
R **(*n*** **= 14,292)**	2622	18.3	11,157	78.1	513	3.6		1701	11.9	10,475	73.3	2116	14.8	
**SES**														
L (*n* = 3865)	1147	29.7	2660	68.9	58	1.4		507	13.1	2658	68.8	700	18.1	
M (*n* = 24,970)	3996	16.0	20,561	82.3	413	1.7	< 0.001*	2200	8.8	17,342	69.5	5428	21.7	< 0.001*
H **(*n*** **= 4315)**	508	11.8	3000	69.5	807	18.7		0	0.0	3213	74.5	1102	25.5	

*: significant *L* Low, *M* Middle, *H* high

**Table 4:** Distribution of anemia and anemia with stunting regarding demographic data among the studied participants

	Anemia				OR (CI95%)	Anemia with stunting				OR (CI95%)
	Yes (No. = 8619)		No (No. = 24,531)			Yes (No. = 3282)		No (No. = 29,868)		
	no	%	No	%		no	%	no	%	
**Age (Y)**										
< 9 (*n* = 21,989)	6011	27.3	15,978	72.7	*1.23 (1.17–1.30)	1945	8.8	20,044	91.1	**1.0**
≥ 9 (*n* = 11,161)	2608	23.4	8553	76.7	**1.0**	1337	12.0	9824	88.0	*1.40 (1.30–1.51)
**Sex**										
Boy (*n* = 17,143)	3810	22.2	13,333	77.8	**1.0**	1457	8.5	15,686	91.5	**1.0**
Girl **(*n*** **= 16,007)**	4809	30.0	11,198	70.0	*1.50 (1.43–1.58)	1825	11.4	14,182	88.6	*1.39 (1.29–1.49)
**Residence**										
Urban (*n* = 18,858)	3841	20.4	15,017	79.6	**1.0**	1467	7.8	17,391	99.2	**1.0**
Rural **(*n*** **= 14,292)**	4778	33.4	9514	66.6	*1.96 (1.87–2.06)	1815	12.7	12,477	87.3	*1.72 (1.60–1.85)
**SES**										
Low (*n* = **4315**)	1493	34.6	2822	65.4	*2.54 (2.29–2.82)	744	17.2	3571	82.8	*3.32 (2.85–3.88)
Moderate (*n* = 24,970)	6459	25.9	18,511	74.1	*1.67 (1.53–1.83)	2310	9.3	22,660	90.7	*1.63 (1.41–1.87)
High **(*n*** **=** 3865**)**	667	17.3	3198	82.7	**1.0**	228	5.9	3637	94.1	**1.0**

*: significant *L* Low, *M* Middle, *H* high

## Discussion

In this study, the prevalence of short stature was about 17%. It is much lower than figures reported in cross-sectional studies conducted locally and internationally [26, 27]. In 2015, the estimated prevalence of stunting among school aged children in Africa and Asia was 37 and 23% respectively [28]. Reduction in the prevalence of stunting in recent years shows that substantial improvements are possible as a result of socioeconomic changes along with specific infection control and dietary interventions. This also consistent with United Nations Decade of Action on Nutrition (from 2016 to 2025) aiming to decrease all types of malnutrition [29]. But this needs greater and ongoing efforts to reach low prevalence like countries as turkey [30]. The significant high prevalence of stunting among males agrees with some previous studies [30, 31], and disagrees with others [32]. In the current study, increased stunting percentage among males and children aged ≥9 years old; can be explained by the emergence of child labor and its negative effects on their health. The demographic analysis of the results showed higher prevalence of stunting among children with low SES and of rural areas residency. This finding agrees with Sharaf et al. [26] The present study assessed causes of short stature and revealed that non pathological causes were the highest in prevalence and stratified as familial, constitutional, and idiopathic. The hallmarks of familial cause were; an appropriate bone age for chronologic age with normal growth velocity, and predicted adult height was appropriate to the family, in Contrast to constitutional cause which characterized by bone age delay with normal growth velocity and predicted adult height was appropriate to the family, they mostly have relative or parents with late puberty [33]. This agrees with Hussein et al. in Egypt who presented the causes as; non pathological variants represented 61.6% and most frequent pathological causes were GHD and hypothyroidism and Velayutham K et al. who showed that familial cause was the most common etiology [22, 34]. but it disagrees with Colaco et al. and Bhadada et al. who reported the prevalence of normal variants to be 20.5 and 15.9% respectively [35, 36]. The present study showed that the prevalence of GHD as a pathological cause to short stature was nearly the same as that measured in Hussein et al., Velayutham et al. and Bhadada et al. [22, 34, 36] while the prevalence of hypothyroidism was much lower than that measured in Velayutham et al. and Bhadada et al. [34, 36], which can be explained by the strict and successful neonatal screening program and availability of thyroid investigations in Egypt. In the present study malnutrition caused stunting in 6.8% of the sample and this is relatively near to reports from other developing countries [36]. The high prevalence of children with short stature rather than malnourished stunted ones; showing that growth in height isn’t dependent only on the extent and nature of the diet and stunting is not a synonym of malnutrition [9]. In contrast, other studies didn’t report malnutrition as a cause of stunting, because they conducted the study on children in tertiary centers referred from other hospitals where they may have received nutritional intervention [22]. Celiac disease despite representing a low percentage in comparison to other causes, but it is very important to be involved in the primary investigations of stunting, especially that celiac disease was thought, incorrectly, to be rare in Egypt [8, 37]. This finding is closer to Abu-Zekry et al. but far from Assiri et al. [37, 38] In our study prevalence of turner syndrome was 0.3% among studied group agrees with other study [22]. Higher prevalence of turner syndrome was reported in other studies conducted on referred children to tertiary care centers or endocrine clinics which mayn’t appropriate to be generalized [39]. Turner syndrome may be presented by stunting without overall stigmata, so it is recommended to include karyotyping in severely stunted female with unexplained growth failure [40]. Underweight prevalence was 8.2% among studied children which is higher than reports from other national studies in high income countries like United Kingdom but equal to that in low SES countries [41, 42]. SES and rural-urban inequalities in underweight were observed in our study and also have been highlighted by many studies [43–45]. Inequality of income between rural and urban households explains most of the malnutrition gap [44]. Understanding of the nature and the underlying factors behind urban-rural health inequalities may help in designing effective measures for improving population health. Present study reported that about 21.8% of the children were overweight and obese, it is much close to the global prevalence which has risen dramatically from just 4% in 1975 to over 18% in 2016 [7]. Also there is a great improvement in the levels of overweight in comparison to Egypt Demographic and Health Survey 2015 which showed overweight prevalence 35 and 36.6% in males and females respectively in the same age group [18]. The present study reported that children from families with sufficient income and higher SES have significantly more positive deviation from mean BMI for age Z-score compared with other children. This phenomenon was found also in developing countries, where fatness of children is related to wealth and poor children are struggling with under nutrition [46]. This relation was found to be more complex as in United States and United Kingdom low SES families have higher risk of obesity and overweight [47, 48]. The high percentage of both under nutrition and overweight was explained by the nutritional scenarios of the developing countries due to socio-economic and demographic transition, dietary habits, lifestyle modification and increasing risks of non-communicable diseases [2]. The present study found that 26% of the children suffered from anemia. It is considered in the moderate category according to classification of public health significance of anemia in populations [49]. This level is close to reported data from WHO, where 25.4% of children suffered from anemia globally at the same age group but higher than report from demographic health survey 2015 in Egypt [49, 18]. Concurrent anemia and stunting represented 9.9% of studied group. This agrees with results from some middle income countries [50]. On the other hand; this is much less than reported from Ethiopia [17]. The high level of co-occurrence of anemia and stunting is highly concerning because each of them are of significant consequences and their co-occurrence is more threating to the children health. β-thalassemia is a common hematologic disorder in the Mediterranean basin, particularly in Egypt, it has been the most common type of genetic anemia with a carrier rate of about 10% [51]. Chronic anemia in general has direct and indirect effects on growth. So, further studies are needed to verify causes of anemia in stunted children in Egypt. Present study showed that anemia and anemia with stunting were significantly higher among children from rural areas and who had low SES and this finding is consistent with Egypt health and demographic survey and Abdel Fatah and Nofal, 2012 which can be explained by higher risk of parasitic infestation, low health services and less iron supplementation [18, 52]. Also Anemia was found to be less frequent among boys which agrees with demographic and health survey 2015 [18].

## Conclusion

This national study reported high prevalence of short stature in addition to underweight, overweight and obesity which characterize the double burden of malnutrition of the low income and developing countries. Based on these results we recommend integration of governmental and non-governmental efforts through health policy and decision makers to achieve the optimal nutrition for populations and individuals. Anemia showed high concurrence with stunting in low SES and rural categories which indicates the great battle against malnutrition. Familial and constitutional causes of short stature were the most prevalent among stunted children. GHD, hypothyroidism, malnutrition, and celiac disease are treatable and frequent conditions that must be considered.

## Data Availability

The datasets used and/or analysed during the current study are available from the corresponding author on reasonable request. (kerosmn1@gmail.com).
